# Clinical Remarks on Vasectomy or Division of the Vasa Deferentia for Some Forms of Vesical Fistulæ Associated with a Large Prostate

**Published:** 1897-04-03

**Authors:** Reginald Harrison

**Affiliations:** Surgeon to St. Peter's Hospital


### CLINICAL REMARKS ON VASECTOMY OR DIVISION OF THE VASA DEFERENTIA FOR SOME FORMS OF VESICAL FISTULÆ ASSOCIATED WITH A LARGE PROSTATE.

By Reginald Harrison, F.R.C.S., Surgeon to St. Peter's Hospital.

One of the disadvantages connected with supracystotomy, for whatever purpose performed, where the prostate is hypertrophied, is the possibility of a permanent fistula remaining, for the reason that urine will always escape from the bladder by the way where the least resistance is offered. This is merely another example of a well-recognised law in hydrostatics.

The following case illustrates how a diminution in the size of the prostate may follow division of the seminal ducts and thus be the means of bringing about the restoration of the natural channel, and consequently the closure of the fistula. Though division of the vasa results in the cessation of sexual desire and power, the age of the patient, taken in conjunction with the prospect of a speedy and effectual removal of a distressing urinary condition, in addition to the other symptoms of an enlarged and obstructive prostate, will, under these circumstances, usually lead to the genital loss being submitted to without hesitation.

Vasectomy, when performed in accordance with the practice adopted in the following instance, is, I believe and have found, free from risks of all kind. The division of both vasa at the same time is not to be recommended in any instance.

The case was that of a gentleman, aged sixty-six, who, on developing an enlarged prostate, was unable to pass renal calculi composed of uric acid, and on two occasions in 1891 I had to crush these, and remove them by the wash bottle. In the course of the following year he suffered much from cystitis, probably more connected with his prostate than with his calculous tendency. In 1892, when absent from London, his symptoms became so urgent that his medical attendant opened the bladder above the pubes, and removed a phosphatic concretion. This operation was thus far successful, but the suprapubic wound remained permanently open until September of last year (1896). Though he did not develop more stone, his prostate and suprapubic fistula became a constant source of pain and annoyance to him. Early in 1894 he again came under my care for his urinary symptoms. The prostate was very large and hard, and there was a suprapubic fistula through which the whole of the urine passed, with the exception of a small amount which he drew off with a catheter. All kinds of expedients having failed to close the fistula, in January, 1894, I opened it up and explored the bladder. There was no stone, but some mortar-like phosphates. The prostate was large, and the middle lobe prominent. I removed the latter, and having freshened up the sinus, closed it with sutures, and retained a rubber catheter in the bladder along the urethra. However, no good came out of this worth mentioning, and the patient's condition was, in the course of a short time, in no way improved. After again waiting and trying other means, with catheters and sutures, and having regard to the condition of the prostate as the primary cause of the fistula by obstructing normal micturition, in February of last year (1896) I resected one vas, and the other in the following May.

By September, and without any other treatment, the fistula had permanently closed, and micturition was again normal, after an interval, to my knowledge, of over three years. This I found, by examination from the rectum and with a catheter, associated with a considerable diminution in the size and sensitiveness of the prostate. Additional interest is attached to this observation, as a partial prostatectomy was practised over two years previously.

The patient writes me (March, 1897) that his fistula remains soundly closed, the process of micturition is normal and independent of catheterism, he has recently increased nearly a stone in weight, and can walk six miles or more daily with pleasure. It must be remembered that this was a suprapubic fistula, which had been in existence for over three years, and had resisted every other mode of treatment that had been tried.

I have tried various methods for resecting the vas. The simplest appears to consist in exposing the spermatic cord as it joins the scrotum by a linear incision over it about an inch in length. The vas is readily made out and protruded towards the incision by pressure from behind, between the finger and thumb. It can then be easily seized with a pair of Spencer Wells' clamp forceps and brought to the surface, where it is cleaned by a little dissection and a blunt hook or aneurism needle passed beneath it. A loop is included by a silk ligature and the free portion removed by scissors. To ligature the duct is insufficient, it being necessary to resect a portion of it. After the loop has thus been removed the stump is returned with the ligature cut short, and the little wound is then closed with a suture or two. Union usually takes place in the course of a few days. In this way the operation can be performed quickly and almost bloodlessly.

